# A Specific Peptide with Calcium-Binding Capacity from Defatted *Schizochytrium* sp. Protein Hydrolysates and the Molecular Properties

**DOI:** 10.3390/molecules22040544

**Published:** 2017-03-29

**Authors:** Xixi Cai, Qian Yang, Jiaping Lin, Nanyan Fu, Shaoyun Wang

**Affiliations:** 1 The Key Laboratory of Analysis and Detection Technology for Food Safety of the MOE, College of Chemistry, Fuzhou University, Fuzhou 350108, China; caixx_0123@163.com (X.C.); ifmb2016@126.com (Q.Y.); nanyan_fu@fzu.edu.cn (N.F.); 2 Institute of Food and Marine Bio-Resources, College of Biological Science and Technology, Fuzhou University, Fuzhou 350108, China; kathleen369@163.com

**Keywords:** *Schizochytrium* sp., defatted protein hydrolysate, calcium-binding peptide, mechanism, cellular uptake

## Abstract

Marine microorganisms have been proposed as a new kind of protein source. Efforts are needed in order to transform the protein-rich biological wastes left after lipid extraction into value-added bio-products. Thus, the utilization of protein recovered from defatted *Schizochytrium* sp. by-products presents an opportunity. A specific peptide Tyr-Leu (YL) with calcium-binding capacity was purified from defatted *Schizochytrium* sp. protein hydrolysates through gel filtration chromatography and RP-HPLC. The calcium-binding activity of YL reached 126.34 ± 3.40 μg/mg. The calcium-binding mechanism was investigated through ultraviolet, fluorescence and infrared spectroscopy. The results showed that calcium ions could form dative bonds with carboxyl oxygen atoms and amino nitrogen atoms as well as the nitrogen and oxygen atoms of amide bonds. YL-Ca exhibited excellent thermal stability and solubility, which was beneficial for its absorption and transport in the basic intestinal tract of the human body. Moreover, the cellular uptake of calcium in Caco-2 cells showed that YL-Ca could enhance calcium uptake efficiency and protect calcium ions against precipitation caused by dietary inhibitors such as tannic acid, oxalate, phytate and metal ions. The findings indicate that the by-product of *Schizochytrium* sp. is a promising source for making peptide-calcium bio-products as algae-based functional supplements for human beings.

## 1. Introduction

Marine microorganisms are showing more and more advantages as major sources for the production of bioactive substances due to their short cycle, high yield and ease of cultivation on a large scale compared with plants [1]. Numerous studies have focused on the extraction of lipids and small molecule substances such as pigments by using organic solvents [1,2]. *Schizochytrium* sp., also known as *Schizochytrium aggregatum*, belonging to the marine fungi, possesses a large number of bioactive substances beneficial to humans, such as unsaturated fatty acids, pigments, proteins [2,3]. *Schizochytrium* sp., as an oil-producing microorganism, and was extensively reported to contain great amount of docosahexaenoic acid (DHA), which is a type of long-chain ω-3 polyunsaturated fatty acids incorporated into the developing brain [2,3,4]. After being used for lipid extraction with organic solvent, the remaining *Schizochytrium* sp. by-products, containing about 41% protein, are usually used for biological bait or just discarded as industrial waste [5]. Efforts are needed in order to transform these biological wastes into value-added bio-products. Thus, the utilization of protein recovered from the defatted by-products presents an opportunity for application in pharmaceutical and food ingredients. Preparation of bioactive peptides from proteins through enzymatic hydrolysis has been a hot topic [6,7,8].

Calcium is an essential inorganic nutrient in the human body and takes part in a lot of important physiological procedures. Calcium deficiency can bring about osteoporosis, hypertension and intestinal cancer [9]. Thus, the intake of dietary calcium has become a general requirement for human beings [10,11]. Ionized calcium has been considered as the prime dietary calcium supplement for humans over the years [12]. However, intestinal absorption of ionized calcium could be easily affected by the presence of dietary factors, such as tannins, phytates, oxalate and other divalent metal ions [13]. Organic calcium supplements show superiority in this aspect. Casein O-phosphopeptides (CPPs) derived from dairy protein are extensively used as mineral carriers nowadays, and present appreciable effect in accelerating calcium absorption [14,15,16]. Other calcium-binding peptides, such as soybean protein hydrolysates, whey protein hydrolysates and serum protein hydrolysates, were also reported to be capable of promoting calcium uptake [17,18,19].

The objective of this study was therefore to isolate specific calcium-binding peptides from defatted *Schizochytrium* sp. protein hydrolysate (SPH), explore their possible chelating mechanism and determine the cellular uptake of calcium. The results of the present study could provide a new train of thought for comprehensive utilization of *Schizochytrium* sp. biomass in order to improve the economic feasibility and nutritional value, suggesting the potential for making microorganism peptide-calcium chelates a functional bio-product for human beings.

## 2. Results and Discussion

### 2.1. Purification of Calcium-Binding Peptide

**Figure 1:**
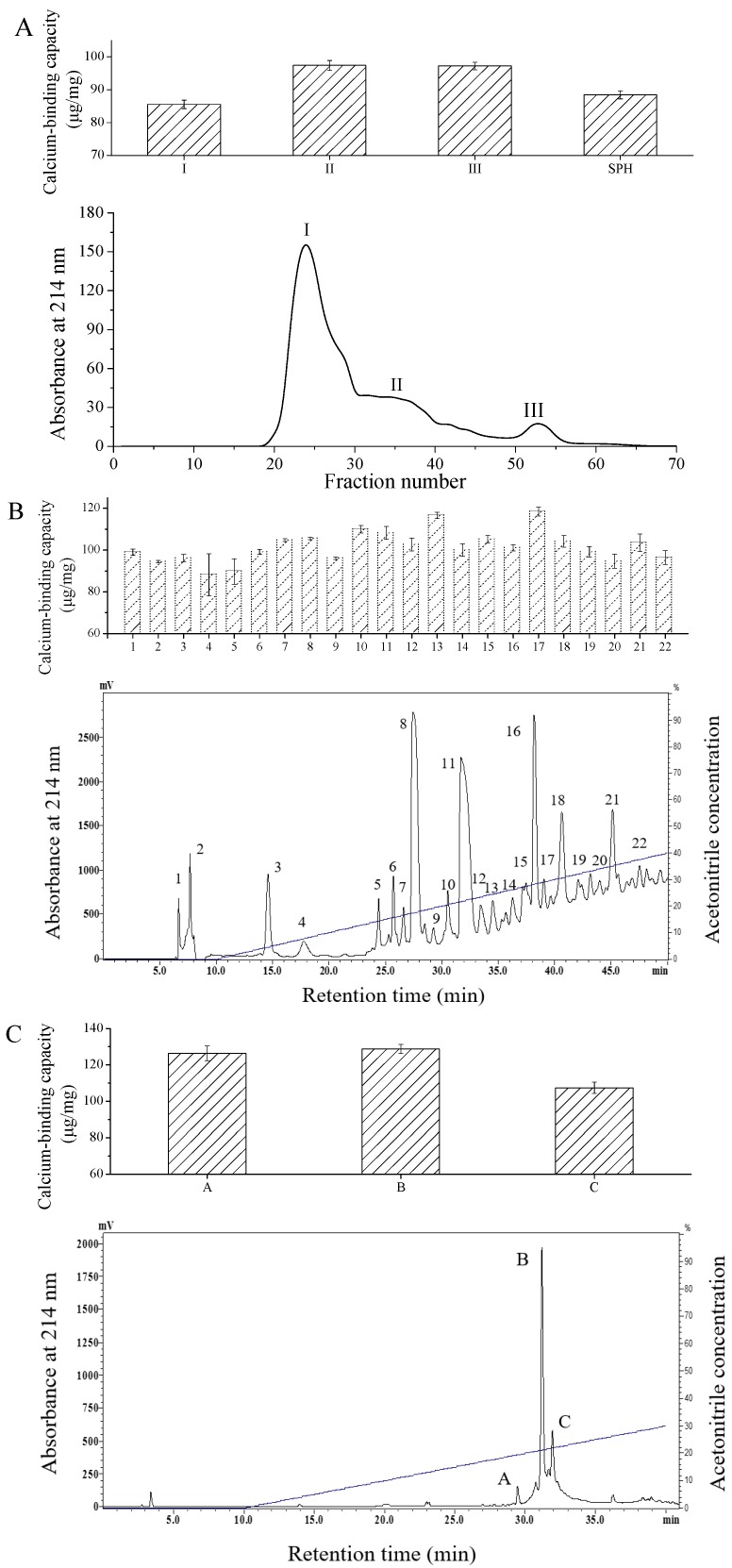
Chromatography elution profiles and calcium binding capacities of calcium-binding peptides. (**A**) Sephadex G-25 gel filtration chromatography of SPH; (**B**) Semi-preparative C18 RP-HPLC of fraction III; (**C**) RP-HPLC of fraction 17 from semi-preparative HPLC.

SPH exhibiting calcium-binding capacity was prepared through stepwise enzymatic hydrolysis. Nanocomposites of SPH chelated with calcium ions were fabricated and characterized by our group in previous study [5]. For the purpose of preparation of peptides with high calcium-binding capacity, SPH was further purified by chromatography. As shown in Figure 1A, SPH was divided into three size-dependent fractions through Sephadex G-25 chromatography. The soluble fraction III with lowest molecular weight and higher calcium binding capacity was collected. This result was in accordance with previous reports showing that peptides with lower molecular weight exhibited higher metal chelating activity [20,21,22]. Fraction III from Sepahdex G-25 was then subjected to semi-preparative C18 RP-HPLC (Figure 1B). Among the 22 distinct fractions, fraction 17 showed the highest chelating capacity and was further purified by analytic RP-HPLC. Finally, a peptide with calcium-binding activity of 126.34 ± 3.40 μg/mg (fraction A) was collected and lyophilized for identification (Figure 1C). Through stepwise chromatography, the calcium-binding capacity of fraction A was enhanced by 42.86% compared to SPH (Table 1).

### 2.2. Identification of the Calcium-Binding Peptide

**Figure 2:**
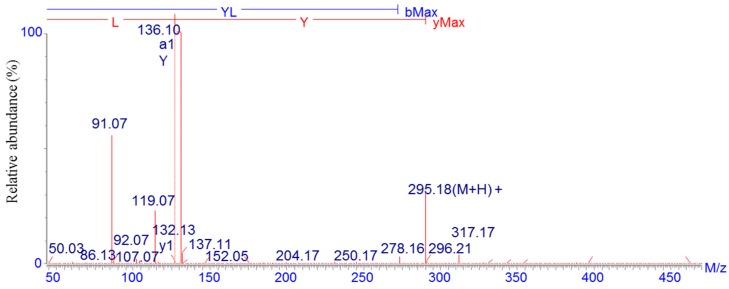
Identification of the amino acid sequence of the calcium-binding peptide using LC-ESI-MS/MS.

The amino acid sequence of fraction A was determined to be Tyr-Leu (YL) with a molecular weight of 294.18 Da using LC-ESI-MS/MS (Figure 2). Calcium-binding peptides from various sources with different molecular weight and sequences have been isolated. A peptide purified from *Chlorella* protein hydrolysates with a calcium binding activity of 0.166 mM and MW of 700.48 Da was reported by Jeon et al. [23]. Two Ca-binding peptides from Alaska pollack backbone and hoki frame hydrolysates were isolated by Jung et al., both of which contained Tyr and Leu residues [24,25]. Tyr, detected in the Ca-binding domain of actin and calmodulin with EF-hand motif, is known as one of the major sites of phosphorylation and metal cation chelating [26]. Moreover, the hydrophobic amino acid Leu was also favorable to calcium binding [14].

### 2.3. Ultra-Violet Spectroscopy Analysis

**Figure 3:**
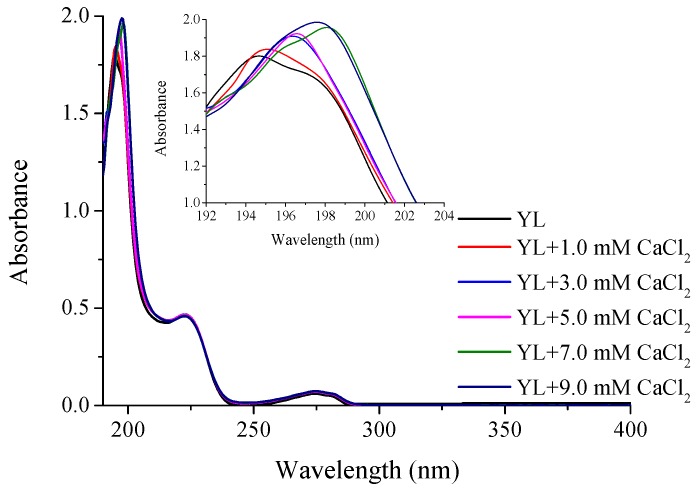
Ultra-violet spectra of YL with different CaCl_2_ concentration.

As shown in Figure 3, the ultraviolet absorption spectra of YL and YL-Ca chelate presented obvious differences. YL exhibited a maximum absorption peak at about 194.5 nm, the intensity of which increased from 1.806 to 2.001 with the increase of calcium concentration, showing hyperchromic effect and redshift phenomenon. These results were in accordance with previous reports that chromophore groups (-C=O, -COOH) and auxochrome groups (-OH, -NH_2_) generate polarizing changes during the chelation of ligand groups and calcium ions [27,28]. Furthermore, both YL and YL-Ca chelate had a specific absorption peak at 280 nm with the same intensity, indicating that phenolic hydroxyl group of Tyr was not involved in the chelation and remained unchanged because of the steric hindrance of the benzene ring. Therefore, the results of UV spectroscopy indicated that nitrogen atom of -NH and -NH_2_ and oxygen atom of -C=O and -COOH might be the functional binding sites in the chelating reaction.

### 2.4. Fluorescence Spectroscopy Analysis

**Figure 4:**
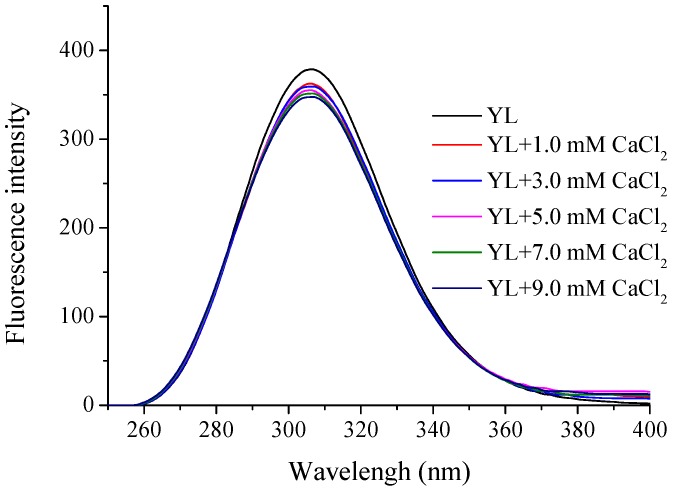
Fluorescence spectra of YL with different CaCl_2_ concentrations.

Tryptophan could generate endogenous fluorescent spectroscopy at excitation wavelength of 280 nm. As shown in Figure 4, with the addition of calcium, the intensity of endogenous fluorescence at 310 nm decreased markedly from 379.876 to 340.312, which meant that calcium ion could be chelated by aromatic amino acids and lead to fluorescence quenching. Obvious endogenous fluorescence quenching appeared as soon as 1.0 mM of CaCl_2_ was introduced. However, with the increase of CaCl_2_, the declining extent of endogenous fluorescence gradually weakened, indicating that changes in the fluorescence intensity occurred when CaCl_2_ chelated with the peptide and overdose free calcium made no difference [29]. Furthermore, Wu et al. proved that reduced fluorescence intensity was a classic indicator of peptide folding when ferrous ions chelated with sturgeon protein peptide, and ferrous ions closed to Tyr residues in the folding process [30]. Hence, the result demonstrated that the calcium ions chelated with YL might cause peptide folding and form compact structure, which contributed to fluorescence quenching [31,32].

### 2.5. Fourier Transform Infrared Spectroscopy (FTIR) Measurement

**Figure 5:**
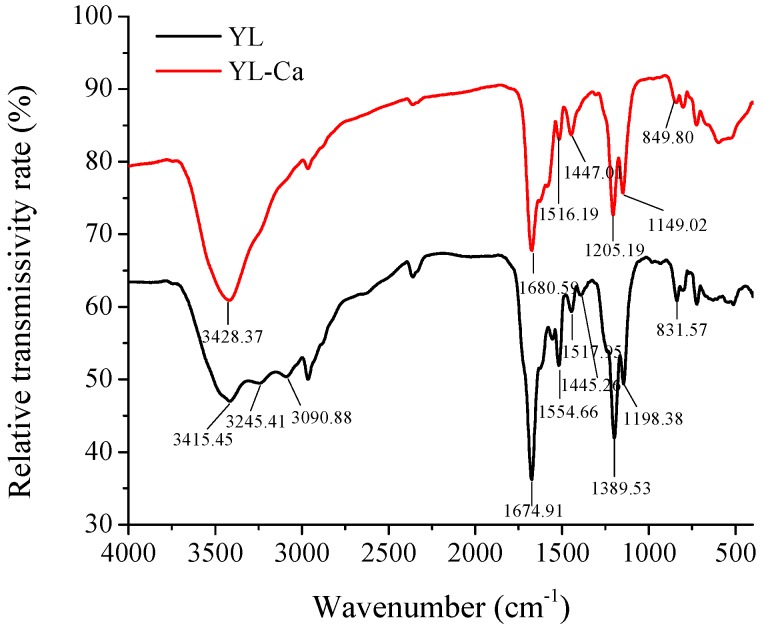
FTIR spectra of YL and YL-Ca chelate.

FTIR could be utilized to observe the functional group changes and reflect the transformation of organic ligand groups after calcium ions are chelated with peptides [33,34]. As shown in Figure 5, the specific absorption peak of the amide-A band moved from 3415.45 cm^−1^ to 3428.37 cm^−1^ and the intensity increased simultaneously when calcium was chelated with YL [35]. The absorption peak intensity of YL at 1554.66 cm^−1^, corresponding to the amide II band vibration, was reduced after the YL-Ca chelate was formed, which could be explained by the fact that the calcium ions chelation induced C-N stretching vibration and N-H bending vibration. Additionally, in the fingerprint region, the intensity of the absorption peak at 1198.38 cm^−1^ increased and shifted to 1149.02 cm^−1^ when YL chelated with calcium ions, indicating that YL bound with calcium ion and formed C-O-Ca [5]. The peak at 831.57 cm^−1^ shifted to 849.80 cm^−1^ and the intensity increased, which was attributable to the increasing electron cloud density of O=C-NH_2_ and the decreasing electron cloud density of N-H when chelation occurred. Wang et al. reported that carboxyl group (-COO-) with negative electricity was potential binding site, and amino group (-NH_2_) and imino group of peptide bond (-NH) were also likely to involve in formation of chelate [36]. Therefore, the results of FTIR indicated that nitrogen atoms of amino group and oxygen atoms of carboxyl group were relevant to the chelation.

### 2.6. Thermal Stability

**Figure 6:**
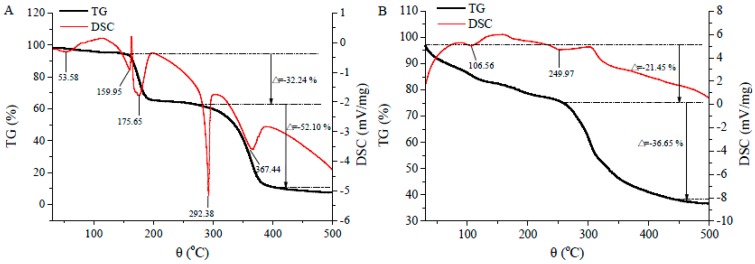
Typical TG-DSC thermograms of (**A**) YL and (**B**) YL-Ca chelate.

**Figure 7:**
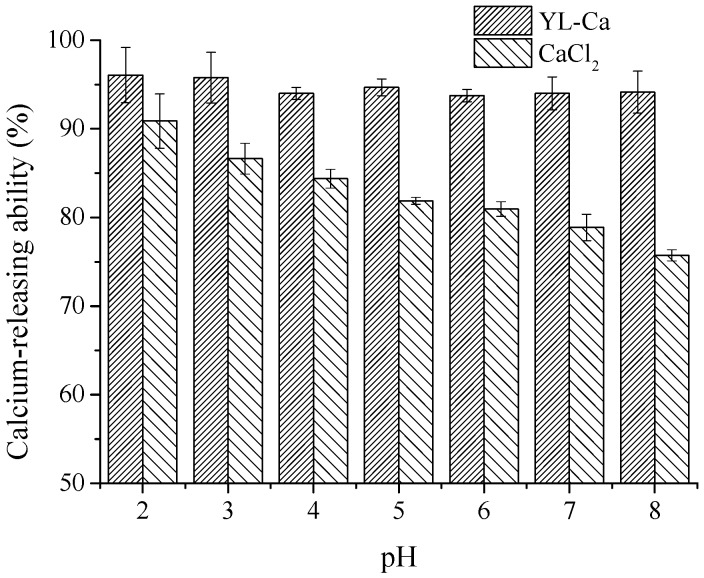
Calcium-releasing percentage of YL-Ca chelate and CaCl_2_ at different pH.

Thermogravimetry-Differential Scanning Calorimetry (TG-DCS) analysis was used to explore the thermodynamic properties and further to evaluate the thermal stability of the material. As the results in Figure 6 show, there were distinct differences of peak temperatures between dipeptide YL and YL-Ca chelate. As shown in Figure 6A, the TG curve presented that the thermal decomposition reaction of YL was divided into two stages and it completely lost 84.34% of the weight in the whole process. At the thermal transition temperatures of 53.38 °C and 159.95 °C there existed endothermic peaks in the DSC curve of Figure 6A, but a weight loss was not apparent in the TG curve, which could be explained by the volatilization of the water in YL during the heating process. Besides, the thermal transition temperatures of the thermal decomposition reaction at 175.65 °C, 292.38 °C and 367.44 °C, were accompanied by obvious weight losses of 32.24% and 52.10%, respectively. The phenomena of endothermic peaks and the accompanying weight loss illustrated that the C-N bonds, in different positions of YL, were destroyed during the heating process [31]. However, the endothermic peaks of YL-Ca significantly shifted to 106.56 °C and 249.97 °C after chelation (Figure 7B). Meanwhile, TG curve of YL-Ca chelate exhibited a weight loss rate of 58.10%. The obvious difference of temperature of constant weight indicated that YL-Ca chelate performed more stable and better thermostability than YL.

### 2.7. Calcium-Releasing Percentage Analysis

The calcium-releasing percentages of YL-Ca chelate and CaCl_2_ at different pH values were measured and the result presented in Figure 7. The calcium-releasing percentage of YL-Ca chelate was always apparently higher than that of CaCl_2_ at pH 2.0–8.0, and it maintained a relatively stable value of about 95% as well. However, CaCl_2_ exhibited obviously diminishing tendency from 90% to 75% at the eventual pH value of 8.0, which could be deduced that the free calcium ions and OH^−^ formed precipitates and led to a decline in the percentage. The pH value in the human intestinal tract is approximately pH 7.2, and YL-Ca chelate had higher solubility and better bioavailability in the gastrointestinal tract, which implied that YL-Ca chelate could be effectively absorbed and transported by intestinal epithelial cells than CaCl_2_ [37].

### 2.8. Cellular Uptake of Calcium in Human Intestinal Cell Lines Caco-2

**Figure 8:**
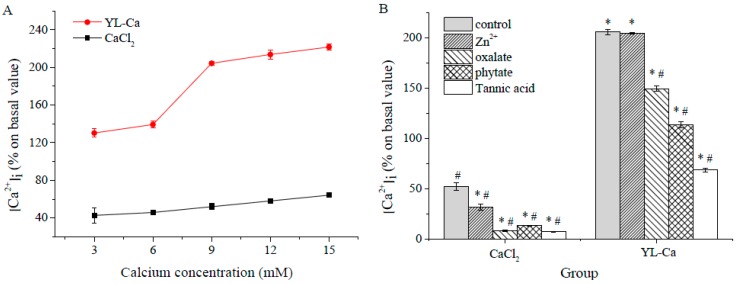
Cellular uptake of YL-Ca chelate and CaCl_2_ in Caco-2 cell model. (**A**) Cell uptake of YL-Ca chelate in Caco-2 cell by Fluo-3-AM loading for fluorescence analysis; (**B**) Effect of YL-Ca chelate on cellular uptake of calcium under the action of dietary inhibitors. The concentration of calcium was 10 mM and tannic acid/Ca, oxalate/Ca, phytate/Ca or Zn/Ca = 20:1. * Statistical significance *p* < 0.05, compared with CaCl_2_ control group. ^#^ Statistical significance *p* < 0.05, compared with YL-Ca control group.

The cytotoxity effect of YL-Ca was determined by MTT assay and verified that YL-Ca did not inactivate Caco-2 cell at calcium concentration of 0–15 mM (data not shown). For the uptake studies, Caco-2 cells were pre-incubated with YL-Ca chelate in different concentrations with CaCl_2_ as control. Changes of the intracellular calcium content were measured by fluorescence spectrometry by using Ca^2+^ binding dyes Fluo-3-AM. The effect of YL-Ca on Caco-2 cell calcium uptake efficiency increased dose-dependently and then approximately trended to stable when the calcium concentration reached 9 mM according to results in Figure 8A. Besides, the uptake-enhanced effects of YL-Ca were more than three times that of CaCl_2_ at the same test calcium concentration.

Previous reports have shown that peptides such as desalted duck egg white peptides, soybean protein hydrolysates and CPPs might act as calcium carriers and interact with the plasma membrane to transport calcium to the cytosol and significantly promote calcium uptake [17,38,39].

To evaluate whether typical inhibitors from food would affect the uptake of calcium chelated by YL, well-established inhibitors like tannic acid, oxalate, phytate and zinc ions were chosen to evaluate their effect on calcium uptake with CaCl_2_ as control. As expected, YL could protect calcium uptake from being affected by inhibitors. The addition of zinc ions, oxalate, phytate and tannic acid severely decreased the calcium uptake efficiency of CaCl_2_ by 39.7%, 84.4%, 74.9% and 86.6%, respectively. However, the calcium uptake efficiency of YL-Ca was 6.5, 18.4, 8.7 and 9.8 times higher than CaCl_2_, respectively, under the same experimental conditions (Figure 8B). Furthermore, the addition of Zn ions had no impact on calcium uptake efficiency of YL-Ca.

Oxalate and phytate are abundant in vegetables and bran could greatly inhibit calcium uptake due to the formation of insoluble and indigestible complex [13,40]. Tannin is another dietary factor belonging to the polyphenol compound class that exhibits extremely strong metal ions complexing and protein degeneration actions, also inducing the precipitation of metal ions [41]. In this study, the addition of tannic acid also decreased the absorptivity of Caco-2 on YL-Ca significantly, which might be due to the fact the peptide was partially denatured by high doses of tannic acid. Despite all of this, the calcium uptake efficiency of YL-Ca was still superior to CaCl_2_ under the same conditions obviously, which might be due to the stronger chelating power of YL than organic phosphate and prevention of calcium precipitation. Divalent metal ions such as zinc and ferrous ions have negative interactions with calcium nutrients and inhibit their uptake since the common receptor for these metal ions DMT1 is located in the intestine [42]. In this study, the inhibition effect of zinc ions on calcium uptake was significantly attenuated with the addition of YL, indicating that YL-Ca might pass through cell membrane through another specific pathways rather than DMT1 receptor. These results demonstrated that YL could prevent a great amount of calcium from being precipitated by certain substances, thus improving calcium uptake. The present study provide powerful evidences for the idea that some proteins/peptides could be considered as mineral carriers because of their ability to bind and solubilize calcium with the possible role in increasing calcium transport across intestinal epithelial cells [43].

## 3. Materials and Methods

### 3.1. Materials

*Schizochytrium* sp. was kindly provided by the Fisheries Research Institute of Fujian, China. The commercial proteases Alcalase (EC. 3.4.21.62, 2.2 × 10^5^ U/g) and Flavourzyme (EC. 3.4.11.1, 7.8 × 10^4^ U/g) were purchased from Novozymes (Copenhagen, Denmark). Sephadex G-25 was the product of Amersham Pharmacia Co. (Uppsala, Sweden). Methanol and acetonitrile used in liquid chromatography were of HPLC grade. All of the other chemicals and solvents were of analytical grade and commercially available.

### 3.2. Extraction of Schizochytrium sp. Protein

The *Schizochytrium* sp. protein (SP) was prepared through alkali extraction and isoelectric point precipitation. The defatted *Schizochytrium* was ground into powder and sieved through a 50 mesh sieve. *Schizochytrium* powder was suspended in 0.39 M of NaOH solution at 1% solid concentration. Protein was extracted at 90 °C under constant shaking for 30 min. The mixture was centrifuged at 10,000 rpm for 20 min, then the supernatant was adjusted to pH 3.0 by 6 M of HCl solution and stood for 30 min. SP precipitation was collected by centrifugation at 10,000 rpm for 15 min and lyophilized for further enzymatic hydrolysis.

### 3.3. Preparation of SPH

SPH were prepared from SP through stepwise enzymatic hydrolysis. The lyophilized SP was dissolved in distilled water at 1% substrate concentration, and the ratio of Alcalase to SP was 10% (*w*/*w*). The hydrolysis was carried out under constant shaking at pH 9.0 and 50 °C for 8.0 h. The sample solution was heated in boiling water for 10 min to inactivate the protease, and then the pH was adjusted to 6.0. Flavourzyme (enzyme/substrate ratio was 10%, *w*/*w*) was subsequently added to get further hydrolysis at 41 °C for 3.78 h. The solution was heated at 100 °C for 10 min to inactive Flavourzyme and then cooled to room temperature. The SPH in the supernatant was collected by centrifugation at 10,000 rpm for 20 min, and then lyophilized for subsequent purification.

### 3.4. Purification of Specific Calcium-Binding Peptides

Chromatography was employed for the purification of specific calcium-binding peptide. The lyophilized SPH dissolved in deionized water was loaded onto a Sephadex G-25 column (100 × 2.0 cm) and then eluted with deionized water at a flow rate of 0.3 mL/min. The absorbance of the elution was monitored at 214 nm and the calcium-binding capacity of the fractions was determined. The fraction with the highest calcium-binding activity from Sephadex G-25 chromatography was pooled and further purified by semi-preparative reversed phase HPLC on a C18 reversed-silica gel column (Gemini 5 μ C18, 250 × 10 mm; Phenomenex Inc., Torrance, CA, USA). Elution was performed with solution A (0.05% trifluoroacetic acid (TFA) in water) and solution B (0.05% TFA in acetonitrile) with a gradient of 0–40% B at a flow rate of 2 mL/min for 50 min. The most active fraction was further purified by analytic HPLC analysis. Buffers A and B were the same as those used in semi-preparative RP-HPLC. Runs were conducted with a liner gradient of 0–30% solvent B at a flow rate of 1 mL/min.

### 3.5. Identification and Synthesis of Purified Calcium-Binding Peptide

The molecular mass and amino acid sequence of the purified calcium-binding peptide were determined using liquid chromatography-electrospray ionization/mass spectrometry (LC–ESI–MS/MS, Delta Prep 4000, Waters Co., Milford, MA, USA) over the *w*/*z* range of 300–3000. The purified peptide (YL) was synthesized by GL Biochem Co. Ltd. (Shanghai, China) through solid-phase procedure. The purity of synthesized peptide was 99.22% by HPLC analysis and the structure of peptide was confirmed by mass spectrometry analysis.

### 3.6. Analysis of Calcium-Binding Activity

The calcium-binding activity was measured with *ortho*-cresolphthalein complexone reagent according to the method described by Wang et al. [37] with some modifications. 1 mL of 1 mg/mL peptides was added to the mixture of 1 mL of 9 mM CaCl_2_ and 2 mL of 0.2 M sodium phosphate buffer (pH 8.0) to make a competitive environment. The mixture was stirred at 37 °C for 2 h. The insoluble calcium phosphate salts was then removed by centrifugation at 10,000 rpm for 10 min and the calcium contents in the supernatant were determined by the absorbance at 570 nm after introducing the working solution to the samples.

### 3.7. Structural Characterization of Peptide–Calcium Chelate

#### 3.7.1. Fabrication of Peptide-Calcium Chelate

100 mg of lyophilized peptide was dissolved in 10 mL of distilled water, and CaCl_2_ solution was introduced subsequently to a ratio of peptide to calcium 3:1 (*w*/*w*) at pH 6.0. The reaction solution was placed in a shaking water bath at 140 rpm and 37 °C for 20 min. Peptide-calcium chelate was precipitated after introducing absolute ethanol and collected by centrifugation at 10,000 rpm for 20 min.

#### 3.7.2. Ultra-Violet Spectroscopy

The UV spectra of calcium-binding peptide and its calcium chelate were monitored over the wavelength range from 190 to 400 nm using a UV spectrophotometer (UV-2600, UNICO Instrument Co. Ltd., Shanghai, China). For determinations, 20 μg/mL of peptide solution was prepared and the pH was adjusted to 6.5. Then 0, 0.5, 1.0, 1.0, 1.0 and 1.0 μL of 2 M CaCl_2_ was constantly introduced every 10 min and the UV spectra were recorded.

#### 3.7.3. Fluorescence Spectroscopy

Fluorescence spectra were measured by a Hitachi F-4600 fluorescence spectrophotometer (Hitachi Co., Tokyo, Japan). The excitation wavelength was 285 nm and the emission wavelengths between 250 and 400 nm were recorded. The slit width of excitation and emission was 20 and 30 nm respectively, and the sensitivity was 1.

#### 3.7.4. FTIR Analysis

1 mg of lyophilized sample was subjected to FTIR spectra in 100 mg KBr and the spectra were recorded at room temperature by an infrared spectrophotometer (360 Intelligent, Thermo Nicolet Co., Madison, WI, USA) from 4000 to 400 cm^−1^. For each spectrum, 64 scans were acquired at 4 cm^−1^ resolution.

### 3.8. TG-DCS Analysis

TG-DSC simultaneous thermal analyzer (STA449C, NETZSCH, Bavaria, Germany) was used to analyze the thermostability of the samples. The lyophilized powder samples (5 mg) were set in hermetic pans and heated from 30 to 500 °C with programmed heating rate of 10 °C/min and argon flow rate of 30 mL/min.

### 3.9. Calcium Releasing Assay

The calcium ions releasing percentages of peptide-calcium chelate and CaCl_2_ (50 μg/mL in deionized water) were determined at pH ranges of 2.0–8.0. After incubation under constant shaking at 140 rpm and 37 °C for 2 h, the reaction solutions were centrifuged at 10,000 rpm for 10 min. The calcium content of the supernatant and the total calcium in the solution were measured using a colorimetric method with ortho-cresolphthalein complexone reagent. The calcium-releasing percentage was calculated as follows:

(1) $$\mathrm{Calcium releasing}\;(\%)=\frac{\text{Calcium in supernatant}}{\text{Total calcium in solution}}\times 100$$

### 3.10. Cellular Uptake of Calcium in Human Intestinal Cell Lines Caco-2

#### 3.10.1. Cell Culture

The human colon adenocarcinoma, Caco-2 cells were grown in DMEM supplemented with 15% (*v/v*) FBS, 1% non-essential amino acid, 100 units/mL penicillin and 100 μg/mL streptomycin and maintained at 37 °C in a humidified atmosphere with 5% CO_2_. At 80–90% confluence, cells were seeded on 12-well plastic cell culture clusters at a density of 1 × 10^4^ cells/cm^2^ for 7 days.

#### 3.10.2. Fluorescence Analysis for Cellular Uptake of Calcium

After grown in 12-well plastic cell culture clusters for 7 days, Caco-2 cells were pre-incubated with peptide-calcium chelate, CaCl_2_, at different concentrations, and tannic acid/phytate/oxalate/Zn^2+^ plus chelate, tannic acid/phytate/oxalate/Zn^2+^ plus CaCl_2_ for 1 h, respectively. The cells were then washed with HBSS three times followed by treatment with 10 μM Fluo-3-AM. After incubation for 1 h, cells were washed with HBSS and harvested for analysis by F-4600 FL Spectrophotometer. Intracellular calcium concentrations [Ca^2+^]_i_ are expressed as an increase in fluorescence intensity compared to baseline, which is the original fluorescence intensity without the addition of exogenous calcium.

### 3.11. Statistical Analyses

All data were presented as means ± standard deviations (SDs) in three replicates. Statistical evaluation was carried out with IBM SPSS 17.0 (Chicago, IL, USA). Comparisons of multiple treatment conditions were analyzed using one-way analysis of variance (ANOVA) with Duncan’s test for *post hoc* analysis. A confidence level of *p* < 0.05 was considered statistically significant.

## 4. Conclusions

In summary, a specific Tyr-Leu dipeptide with strong calcium-chelating capacity was purified from defatted *Schizochytrium* sp. protein hydrolysates and the chelating mechanism was investigated. It was shown that calcium ions could form dative bonds with carboxyl oxygen atoms and amino nitrogen atoms as well as the nitrogen and oxygen atoms of amide bonds, inducing conformational changes of the dipeptide and ultimately a new and stable peptide-calcium chelate was formed. The cellular uptake efficiency of YL-Ca was superior to CaCl_2_, suggesting the potential of YL-Ca to be used as functionally nutraceutical additives.

## Figures and Tables

**Table 1:** Chromatography purification and the calcium-binding capacity.

Fraction	Calcium-Binding Capacity (μg/mg)	Activity Enhancement (%)
SPH	88.43 ± 1.25	-
Fraction III from Sephadex G-25	97.25 ± 1.14	9.96
Fraction 17 from semi-preparative RP-HPLC	118.36 ± 2.13	33.84
Fraction A from analytic RP-HPLC	126.34 ± 3.98	42.86
